# Evaluating the credibility of claims and recommendations in educational psychology: a grand challenge

**DOI:** 10.3389/fpsyg.2026.1779841

**Published:** 2026-03-31

**Authors:** Daniel H. Robinson

**Affiliations:** College of Education, The University of Texas at Arlington, Arlington, TX, United States

**Keywords:** artificial intelligence, causation, claims, educational psychology, misinformation

I started my term as Specialty Chief Section Editor for *Frontiers in Psychology: Educational Psychology* (*FIP:EP*) on September 1, 2025. I previously served as editor of *Educational Psychology Review* (2006–2025) and as associate editor of the *Journal of Educational Psychology* (2014–2020). What is most different about my latest editorial role is the sheer number of manuscripts. Among the 34 sections of *Frontiers in Psychology*, the educational psychology section is the largest. In 2025, we received well over 2,000 submissions. For comparison, the *Journal of Educational Psychology* received 837 submissions in 2023.

Beyond taking on a role that requires herculean efforts on the part of everyone involved in the editorial process, I am also becoming more familiar with the type of research that is submitted, the claims the authors make, and the implications for the field. One question has far outweighed others as I get my feet on the ground in my new role: what is the evidence base for claims and recommendations?

This question is not a new one for me by any means. For over 20 years, my colleagues and I have examined trends in the field of educational psychology in an attempt to answer the question (Brady et al., 2023; Hsieh et al., 2005; Reinhart et al., 2013; Robinson et al., 2007). Three themes have emerged from this work: (1) the proportion of empirical articles published in educational psychology journals (*Journal of Educational Psychology, American Educational Research Journal, Cognition and Instruction, Journal of Experimental Education*, and *Contemporary Educational Psychology*) that employ intervention methods has decreased from 47% in 1994 to 25% in 2020; (2) conversely, the proportion that employ observational methods has increased from 43% in 1994 to 66% in 2025; and (3) the proportion of observational articles that include recommendations for practice has increased from 30% in 1994 to 66% in 2020.

An astute reader will have noticed that *FIP:EP* is not listed among the journals we consider to be the Big Five journals in the field. There are two reasons for this exclusion. First, the journal is relatively new, launching in 2010. Second, unlike the other five journals, *FIP:EP* is solely open access, with author publication fees. But now that *FIP:EP* is clearly the most popular educational psychology journal in the world, it is time that we take stock of where we are and how far we have come. Thus, my grand challenge in this editorial is for a similar examination of the *FIP:EP* section.

The latest article in our line of inquiry is from Brady et al. (2023). As of December 22, 2025, this article has been cited 50 times according to Google Scholar and 34 times according to the Web of Science. It seems that questioning the scientific validity of a field elicits a considerable response. I want to challenge the readers of, and contributors to, *FIP:EP* to seriously consider what this means for them.

In a response to the reactions from the field, Robinson and Wainer (2023) wrote:

In educational psychology, we have moved away from a very important component of research—rigor. Instead, we have been seduced by what is simple and easy. The fruits of this latter type of research are rarely useful in providing causal evidence. (p. 82)

As section editor, I want to invite manuscripts that examine whether the articles published in *FIP:EP* have an evidence base that supports their corresponding claims and recommendations.

I also welcome other examinations of *FIP:EP*, including of who has contributed as authors, editorial members, etc. Are women participating at a rate consistent with their increasing involvement in the field as researchers, faculty, and students? I have been involved in similar investigations (e.g., Fong et al., 2022; Griffin et al., 2023) and am convinced that such studies can serve as a healthy audit for the journal.

Besides looking backwards and assessing where we have come as a field, we also need to consider the future. What will be the key issues and new directions in educational psychology? I see two areas that are relevant here. First, how will artificial intelligence (AI) influence how we pursue knowledge? Generative AI has already begun to shape the way we teach, assess, and learn (Mayrath et al., in press). But along with enthusiasm about its potential, there is an accompanying fear that AI will hurt student engagement and learning by providing easy shortcuts and new ways to plagiarize. I expect to see more submissions that address how we can harness AI to augment, rather than replace, student learning. How can AI be used to improve assessment of learning? How will the roles of the instructor change? How will learning materials be different in this age of AI?

Second, getting back to the main point of this editorial, educational psychology must also continue to deal with the abundance of misinformation, pseudoscience, and quackery that appears daily in popular media outlets. We must continue to serve as watchdogs and call out policies that are not supported by scientific research. In the U.S., we have witnessed a resurgence of childhood diseases such as measles that had been eradicated long ago only to resurface due to vaccine hesitancy. There is a level of distrust in science that is reaching an all-time high. In education, we continue to see recommendations to teach to students' learning styles to improve learning despite overwhelming evidence to the contrary (Cleary and Robinson, 2025; Robinson et al., 2022). Other popular educational practices have been recently challenged, including universal design for learning (Boysen, 2024) and growth mindset training (Macnamara and Burgoyne, 2023). Our first obligation to society must be to protect society from our field. Educators look to us for practical solutions to difficult problems. When we publish empirical findings that are accompanied by unwarranted claims and recommendations, we commit a huge disservice by providing more misinformation. Educational psychology needs to continue to identify what does and does not work.
